# HSV-1 and Cellular miRNAs in CSF-Derived Exosomes as Diagnostically Relevant Biomarkers for Neuroinflammation

**DOI:** 10.3390/cells13141208

**Published:** 2024-07-17

**Authors:** Christian Scheiber, Hans C. Klein, Julian M. Schneider, Tanja Schulz, Karl Bechter, Hayrettin Tumani, Thomas Kapapa, Dani Flinkman, Eleanor Coffey, Duncan Ross, Maksims Čistjakovs, Zaiga Nora-Krūkle, Daria Bortolotti, Roberta Rizzo, Modra Murovska, E. Marion Schneider

**Affiliations:** 1 Clinic for Anaesthesiology and Intensive Care Medicine, Ulm University Hospital, 89081 Ulm, Germany; christian.scheiber@alumni.uni-ulm.de (C.S.); julian-2.schneider@uni-ulm.de (J.M.S.); tanja.schulz@uni-ulm.de (T.S.); 2 Department of Neurology, Ulm University Hospital, 89081 Ulm, Germany; hayrettin.tumani@uni-ulm.de; 3 Department of Nuclear Medicine and Molecular Imaging, University Medical Center Groningen, University of Groningen, 9700 RB Groningen, The Netherlands; hanscklein@gmail.com; 4 Research and Education Department Addiction Care Northern Netherlands, 9728 JR Groningen, The Netherlands; 5 Clinic for Psychiatry and Psychotherapy II, Ulm University, 89312 Guenzburg, Germany; karl.bechter@bkh-guenzburg.de; 6 Department of Neurosurgery, Ulm University Hospital, 89081 Ulm, Germany; thomas.kapapa@uniklinik-ulm.de; 7 Turku Bioscience Centre, University of Turku and Åbo Akademi University, 20521 Turku, Finland; dkflin@utu.fi (D.F.); ecoffey@abo.fi (E.C.); 8 Kimera Labs Inc., Miramar, FL 33025, USA; duncan.ross@kimeralabs.com; 9 Institute of Microbiology and Virology, Riga Stradins University, 1067 Riga, Latvia; maksims.cistjakovs@rsu.lv (M.Č.); zaiga.nora@rsu.lv (Z.N.-K.); modra.murovska@rsu.lv (M.M.); 10 Department of Chemical, Pharmaceutical and Agricultural Science, University of Ferrara, Via Luigi Borsari, 46, 44121 Ferrara, Italy; brtdra@unife.it (D.B.); roberta.rizzo@unife.it (R.R.); 11 Laboratory for Advanced Therapeutic Technologies (LTTA), University of Ferrara, 44121 Ferrara, Italy

**Keywords:** CSF exosomes, low-grade inflammation, encephalitis, HSV-1, IL-8, miRNA, neuronal damage, NfL, oxidative stress, psychiatric disease, traumatic brain injury

## Abstract

Virus-associated chronic inflammation may contribute to autoimmunity in a number of diseases. In the brain, autoimmune encephalitis appears related to fluctuating reactivation states of neurotropic viruses. In addition, viral miRNAs and proteins can be transmitted via exosomes, which constitute novel but highly relevant mediators of cellular communication. The current study questioned the role of HSV-1-encoded and host-derived miRNAs in cerebrospinal fluid (CSF)-derived exosomes, enriched from stress-induced neuroinflammatory diseases, mainly subarachnoid hemorrhage (SAH), psychiatric disorders (AF and SZ), and various other neuroinflammatory diseases. The results were compared with CSF exosomes from control donors devoid of any neuroinflammatory pathology. Serology proved positive, but variable immunity against herpesviruses in the majority of patients, except controls. Selective ultrastructural examinations identified distinct, herpesvirus-like particles in CSF-derived lymphocytes and monocytes. The likely release of extracellular vesicles and exosomes was most frequently observed from CSF monocytes. The exosomes released were structurally similar to highly purified stem-cell-derived exosomes. Exosomal RNA was quantified for HSV-1-derived miR-H2-3p, miR-H3-3p, miR-H4-3p, miR-H4-5p, miR-H6-3p, miR-H27 and host-derived miR-21-5p, miR-146a-5p, miR-155-5p, and miR-138-5p and correlated with the oxidative stress chemokine IL-8 and the axonal damage marker neurofilament light chain (NfL). Replication-associated miR-H27 correlated with neuronal damage marker NfL, and cell-derived miR-155-5p correlated with oxidative stress marker IL-8. Elevated miR-138-5p targeting HSV-1 latency-associated ICP0 inversely correlated with lower HSV-1 antibodies in CSF. In summary, miR-H27 and miR-155-5p may constitute neuroinflammatory markers for delineating frequent and fluctuating HSV-1 replication and NfL-related axonal damage in addition to the oxidative stress cytokine IL-8 in the brain. Tentatively, HSV-1 remains a relevant pathogen conditioning autoimmune processes and a psychiatric clinical phenotype.

## 1. Introduction

Autoimmune encephalitis has long been linked to virus infections and reactivation [1]. After primary infection, *herpes simplex virus 1* (HSV-1) replicates and may eventually reactivate in neurons of the central nervous system, triggering an inflammatory response and condition for autoimmunity against NMDA receptors. Active HSV-1 replication further leads to neuronal cell death, whereas non-lytic infection is linked to HSV-1 latency with no tissue damage but with a yet unknown contribution to immune activation [2]. Neuroinflammation and NMDA receptor blockade by autoantibodies may play a role in severe psychiatric disorders like schizophrenia [3]. The etiology of schizophrenia is not fully understood yet, but neuroinflammation is considered an important aspect, as numerously reported by elevated levels of pro-inflammatory cytokines [4]. As possible inducers of neuroinflammation, viral infections have long been proposed to play a role in the development of schizophrenia, and elevated antibodies against HSV-1 were reported in patients with schizophrenia [5,6]. The chronicity of inflammation triggered by the virus might facilitate states of immune insufficiencies and thus lead to the reactivation of other viruses, resulting in a psychiatric phenotype as reported in patients with affective (AF) and schizophrenic (SZ) spectrum disorders [7]. The common denominators of virus reactivation states comprise both intrinsic and exogenous stress factors as described by Marcocci and colleagues [8]. In addition to mental stressors, subarachnoid hemorrhage (SAH) causes massive oxidative stress due to trauma, or rupture of cerebral aneurysms [9,10], and is eventually complicated or even induced by HSV-1 reactivation [11].

For the search for new biomarkers, exosomes, representing a class of membrane vesicles, may contribute significantly Exosomes are easily transported by body fluid circulation, targeting other cells and tissues by phagocytosis and membrane fusion events [12,13]. Among other effects, exosome-guided events explain aspects of communication between the brain and the gut microbiome [14]. Intensive research is elementary to further develop diagnostic and therapeutic potentials by exosomes. Previous reports confirm this approach as a valid and suitable method in other neurological diseases, as well as malignancies [15,16]. Exosomes are highly enriched in microRNAs [17]. miRNAs are small regulatory RNA molecules, able to fine-tune gene regulation by selectively silencing mRNAs [12]. In HSV-1 infection, latency-associated transcripts (LATs) encode viral miRNAs which are antisense to mRNA transcripts of virus-encoded infected cell proteins (ICPs) [18]. Well-defined examples are HSV-1-encoded miR-H2 (reported antisense to ICP0), miR-H6 (antisense to ICP4), miR-H3, and miR-H4 (both antisense to ICP34.5) [18,19]. HSV-1-derived miRNAs were proposed to be suitable to prove the presence of HSV-1 in the brain and selectively distinguish active from latent states of HSV-1 infection. According to previous investigations, the presence of miR-H1 and miR-H27 is linked to productive (active) infection, whereas miR-H2 miR-H3, miR-H4, and miR-H6 would identify states of HSV-1 latency [20,21].

Besides viral miRNAs, cellular miRNAs are relevant for substantiating host–virus interactions, inflammation, and signaling pathways, related to the manifestation of mental disorders [22,23]. Alterations of host miRNAs such as miR-155-5p, miR-21-5p, miR-146a-5p, and miR-138-5p qualify the host’s immune response by their effect to guide signaling. MiR-155-5p is derived from an exon of a non-coding RNA within the *MIR155HG* (MIR155 host gene). The latter was found transcriptionally active at a retroviral integration site in B-cell lymphomas and therefore alternatively named BIC (B-cell integration cluster) [24]. MiR-155-5p is necessary and relevant for interferon-gamma (IFN γ) transcription and the activation of natural killer (NK) cells, macrophages, and Th1 cell polarization, thus promoting pro-inflammatory immunity, also in the context of tumor-related diseases [25]. MiR-146a-5p is derived from the *MIR146A* gene, which does not encode for other sequences besides the miR-146 primary-miRNA (pri-miRNA) (https://www.ncbi.nlm.nih.gov/gene/406938, accessed on 7 July 2024). MiR-146a-5p is highly expressed in TfHs (T follicular helper cells), which activate B cells and promote antibody formation by targeting ICOS (inducible T-cell co-stimulator). In myeloid cells, miR-146a-5p attenuates NF-κB signaling by the inhibition of IRAK and TRAF-6 transcription [25,26]. MiR-21-5p is one of the first identified mammalian miRNAs and is encoded within the coding region of TMEM49 [27]. MiR-21-5p plays an important role in cancer by modulating tumor suppressors such as PTEN [28], but it is also described as a key factor in modulating inflammation [29], e.g., by targeting IL-10 suppressor PDCD4 [30]. Neuronal miR-138-5p targets viral ICP0 and promotes HSV-1 latency [31,32].

The current study addressed the question of whether HSV-1-derived miRNAs are detectable in CSF-derived extracellular vesicles (EVs, including exosomes) of patients with neuroinflammation (AF, SZ, other neuroinflammatory diseases) and brain hemorrhage (SAH patients) and whether host-derived inflammatory miRNA species, the chemokine IL-8, and the neuronal damage marker NfL were linked to HSV-1 latency or active replication. Quality control of exosomal fractions was performed by transmission electron microscopy (TEM), negative staining, and dynamic light scattering (DLS). HSV-1 latency was probed by miR-H2-3p, miR-H3-3p, miR-H4-3p, miR-H4-5p, and miR-H6-3p, while states of HSV-1 replication were judged by miR-H27. Consecutively, this miRNA panel was chosen for distinguishing HSV-1 latency from replication. In addition to their effect on virus transcription, these HSV-1-encoded miRNAs have been reported to influence several cellular pathways, including apoptosis, TGF-β signaling, and neurodegenerative events [21,33]. The results of CSF-derived exosomal miRNA profiling identified miR-H27 as the best discriminative marker for active HSV-1 infection, combined with pro-inflammatory cellular miR-155-5p.

## 2. Materials and Methods

### 2.1. Patients

CSF from *n* = 5 non-neuroinflammatory controls devoid of signs of neuroinflammation was obtained from the Auria Biobank (University of Turku, Finland, project number AB22-8062). CSF from *n* = 5 patients with subarachnoid hemorrhage (SAH) was obtained at the Department of Neurosurgery, Ulm University Hospital (Ethics approval No. 82/07). CSF samples from psychiatric patients refractory to therapy were recruited at the Department of Psychiatry and Psychotherapy II, Guenzburg, Ulm University [34,35]. Inclusion criteria were schizophrenic (SZ) or affective (AF) spectrum disorders (ICD-10 F20-F25 and F30-F33, respectively), and informed written consent (Ethics approvals No. 41/2001, and 17-04/2006). In total, CSF was obtained from *n* = 11 AF and *n* = 6 SZ patients. CSF sampling was completed with *n* = 9 patients with other neuroinflammatory diseases. CSF from patients with acute virus infections suffering from infection-related cognitive dysfunction were grouped as acute virus. Individual CSF specimens were studied within routine clinical examinations for the diagnosis of hemophagocytic lymphohistiocytosis (HLH) [36] or were collected under ethics approval No. 20/10, Ulm University. Detailed information on patient groups is provided in Table 1 and Table S2.

### 2.2. EVs’ and Exosomes’ Enrichment from CSF

The enrichment of EVs was based on previously reported protocols based on ultracentrifugation [37]. Aliquots from fresh CSF samples (2 mL) were immediately centrifuged (140× *g*, 3 min, 4 °C) using a swing-out rotor to sediment intact leukocytes (step 1). The supernatants of step 1 were subjected to a second centrifugation step (12,000× *g*, 5 min, 4 °C) in a fixed-angle rotor (Eppendorf centrifuge, Eppendorf SE, Hamburg, Germany), in order to remove large EVs (microparticles, nucleosomes) (step 2). Aliquots of the supernatant of this second centrifugation step were subjected to ultracentrifugation (174,000× *g*, 1 h, 4 °C) using a Beckman Optima™ (Brea, CA, USA) MAX-E (TLA-55 fixed-angle rotor, www.beckmancoulter.com, accessed on 7 July 2024), to enrich the smallest (exosomal) EV fraction (step 3). The contents of each fraction prepared by step 1–3 centrifugation were quality-checked by DLS and ultrastructural analysis. The ultracentrifuged sediment of step 3 (50 µL) was subjected to dynamic light scattering (DLS) and RNA isolation. An overview of all enrichment procedures is given in Supplementary Figure S2.

### 2.3. Transmission Electron Microscopy (TEM)

Fresh CSF samples (600 µL, containing all cellular components) were centrifuged at 140× *g* for 5 min (similar to step 1) to remove cell fragments and EVs. The sediment was then gently resuspended and fixed with 2.5% glutardialdehyde (grade I, Sigma G-5882, Sigma-Aldrich, St. Louis, MO, USA)/PBS for 20 min, as schematically shown in Figure S2.

Fixed specimens were post-fixed with 1% OsO_4_ in 0.1 M phosphate buffer and 0.1% uranyl-acetate (UA) for post-staining, followed by Epon 812 (Taab Laboratory Equipment, Aldermaston, UK) embedding and ultrathin (80 nm) sectioning using a UC-7 ultramicrotome (Leica Microsystems, Wetzlar, Germany). Ultrathin sections were mounted onto copper grids, dried, and post-stained with lead citrate. Specimens were examined using a JEOL 1400 transmission electron microscope (JEOL GmbH, Freising, Germany, www.jeol.com, accessed on 7 July 2024). 

Highly purified exosomes from mesenchymal stem cells (Kimera Labs Inc, Miramar, FL, USA, Kimeralabs.com, accessed on 7 July 2024) served as controls. These exosome preparations were thawed in an ice-cold water bath and immediately cryo-preserved using glass capillaries (M. Wohlwend GmbH, Sennwald, Switzerland) and frozen in a high-pressure freezer (HPF) Compact 01 (M. Wohlwend GmbH, Sennwald, Switzerland). Samples were freeze-substituted with a substitution medium consisting of acetone with 0.2% osmium tetroxide, 0.1% uranyl acetate, and 5% water. Within 17 h, the temperature was raised exponentially from −90 °C to 0 °C. Following substitution, the samples were kept at room temperature for 1 h and then washed twice with acetone, followed by Epon 812 embedding and polymerization for 72 h at 60 °C. For TEM, 70 nm thick sections were cut from the epoxy resin block using a Leica Ultracut UCT ultramicrotome (Leica Microsystems, Wetzlar, Germany, leica-microsystems.com, accessed on 7 July 2024). The sections were mounted onto copper grids, post-stained with 0.1–0.4% filtered lead citrate in purified water, and imaged at 80 kEV with a Jeol 1400 transelectron microscope (JEOL GmbH, Freising, Germany, www.jeol.com, accessed on 7 July 2024). Stem-cell-derived exosomes were also examined following negative staining protocols. In detail, stem-cell-derived exosomes (1.5 × 10^9^ exosomes/mL) were diluted 1:10 in PBS, and 20 µL of this solution was pipetted onto copper grids, dried, and post-stained with 1% uranyl acetate.

TEM served as a quality control for the identification of membrane-surrounded vesicles. The results of TEM examinations should correspond to EV size distribution by DLS measurements.

### 2.4. Dynamic Light Scattering (DLS)

EV size distributions were determined by dynamic light scattering (DLS) using the Litesizer™ 500 Particle Analyzer (Anton Paar Group AG, Graz, Austria, www.anton-paar.com, accessed on 7 July 2024) and corresponding software (Anton Paar Group AG, Graz, Austria, Anton Paar Kalliope^TM^ Professional, version 2.22.1). EV sediments (50 µL) were diluted 1:10 in DPBS (1X, Thermo Fisher Scientific Inc., Waltham, MA, USA, www.thermofisher.com, accessed on 8 July 2024), and briefly vortexed. Next, 60 µL of the PBS-diluted specimen was analyzed in low-volume cuvettes, UVette^®^ (Eppendorf SE, Hamburg, Germany, www.eppendorf.com, accessed on 7 July 2024). The temperature was set to 25 °C, and the optical filter was selected automatically. The results were documented from a maximum of 60 individual measurements.

### 2.5. RNA Isolation

RNA was isolated from EV sediments (50 µL), using the Maxwell^®^ 16 miRNA Tissue Kit (AS1470, Promega Corporation, Madison, WI, USA, www.promega.com, accessed on 7 July 2024), a Maxwell^®^ 16 Instrument, and the software protocol *simply RNA* (Promega Corporation, Madison, WI, USA, www.promega.com, accessed on 7 July 2024) according to the manufacturer’s protocol. EV sediments were adjusted to a final volume of 200 µL with the homogenization solution (Promega Corporation, Madison, WI, USA www.promega.com, accessed on 7 July 2024) provided by the manufacturer. For RNA elution, 55 µL of nuclease-free water (www.promega.com, accessed on 7 July 2024) was used.

### 2.6. RT-qPCR

For HSV-1-derived miRNAs, 5 µL of total RNA was reverse-transcribed using the TaqMan™ MicroRNA Reverse Transcription Kit (Thermo Fisher Scientific Inc., Waltham, MA, USA, www.thermofisher.com, accessed on 7 July 2024). Synthesis of cDNA was performed according to the manufacturer’s protocol. RT-qPCR was performed with TaqMan probes (Thermo Fisher Scientific Inc., Waltham, MA, USA, www.thermofisher.com, accessed on 7 July 2024) detecting miR-H2-3p (005632), miR-H3-3p (197242_mat), miR-H4-3p (197191_mat), miR-H4-5p (007828_mat), miR-H6-3p (197219_mat), and miR-H27 (469286_mat). For obtaining the most suitable candidate for normalization, the stability of the investigated viral miRNAs was determined by the comparative ΔCT algorithm [38]. This method compares candidate genes pair-wise for all investigated samples and uses the ΔCT values’ standard deviations for each target to derive its stability [38]. This algorithm was recently used in the context of vesicle-derived miRNAs [39]. In our analysis, viral miR-H6-3p was identified as the most stably expressed miRNA species (Table S6, Figure S3) and was used as an endogenous normalization control. Viral miRNA expression was then calculated as fold changes according to the 2^−ΔΔCT^ method [40]. First, a ΔCT value (CT value of the miRNA of interest subtracted by the CT value of viral miR-H6-3p) for every sample and every miRNA was determined. A ΔΔCT value was calculated by subtracting the mean ΔCT value of the control group (Ctrl, implementing all biological replicates) from the ΔCT value of each individual sample. Finally, fold changes are calculated as 2^−ΔΔCT^; in other words, the value doubles with every reduction of a single cycle in ΔCT values [40].

For host miRNAs, 4.5 ng of total RNA were reverse-transcribed using the TaqMan^®^ Advanced miRNA cDNA Synthesis Kit (Thermo Fisher Scientific Inc., Waltham, MA, USA, www.thermofisher.com, accessed on 7 July 2024). Synthesis of cDNA was performed according to the manufacturer’s protocol. RT-qPCR was performed with TaqMan probes (Thermo Fisher Scientific Inc., Waltham, MA, USA, www.thermofisher.com, accessed on 7 July 2024) detecting miR-21-5p (477975_miR), miR-138-5p (477905_miR), miR-146a-5p (478399_miR), miR-155-5p (483064_miR), and *cel*-miR-39-3p (478293_miR). Spiked-in *cel*-miR-39-3p (biomers.net GmbH, Ulm, Germany, www.biomers.net, accessed on 7 July 2024) served as exogenous normalization control. Host miRNA expression was calculated as fold changes according to the 2^−ΔΔCT^ method [40]. First, a ΔCT value for every sample and every miRNA was determined, where ΔCT represents the difference between the CT value of the miRNA of interest and the CT value of spiked-in *cel*-miR-39-3p in the corresponding sample. Next, ΔΔCT was calculated as the difference between the ΔCT value of each sample and the mean ΔCT value of the control group (Ctrl, implementing all biological replicates). Finally, fold changes were calculated as 2^−ΔΔCT^.

All reactions were run on a StepOne™ Real-Time PCR System with corresponding software (StepOne™ software, version 2.3, Thermo Fisher Scientific Inc., Waltham, MA, USA, www.thermofisher.com, accessed on 7 July 2024) according to the respective TaqMan™ Assays protocols (Thermo Fisher Scientific Inc., Waltham, MA, USA, www.thermofisher.com, accessed on 7 July 2024). Runs contained no-template controls (NTCs) for each target. All samples were run in triplicates. For undetectable transcripts, a cycle threshold (C_T_) value of 40 was assumed. CT values between 36 and 40 are used to describe miRNAs which are marginally detected [41].

### 2.7. Quantification of Interleukin-8 (IL-8) and Neurofilament Light Chain (NfL)

Interleukin-8 (IL-8) was quantified by a semi-automated, highly standardized chemiluminescence assay (Immulite^®^1000, Siemens Healthineers AG, Erlangen, Germany, www.siemens-healthineers.com, accessed on 7 July 2024) according to the manufacturer’s protocol. Quantification of neurofilament light chain (NfL) was performed using an automated immunoassay system (Ella^TM^, Bio-Techne Corporation, Minneapolis, MN, USA, www.bio-techne.com, accessed on 7 July 2024) according to the manufacturer’s protocol.

### 2.8. Data Visualization and Statistics

Data visualization and statistical analysis was performed using GraphPad PRISM software (version 9.1.1, www.graphpad.com, accessed on 7 July 2024). EV size distributions were generated by the Anton Paar Kalliope^TM^ Professional software (version 2.22.1, Anton Paar Group AG, Graz, Austria, www.anton-paar.com, accessed on 7 July 2024). TEM served as a quality control for the identification of membrane-surrounded vesicles.

### 2.9. Determination of Herpesvirus Serology

IgG antibodies against HSV-1/-2 were tested in CSF and the corresponding serum samples (Virotech HSV Screen ELISA EC108.00, Virotech Diagnostics GmbH, Dietzenbach, Germany, virotechdiagnostics.com, accessed on 7 July 2024). The results are presented in Table S3 and given as Virotech Units (VUs). According to the manufacturer’s protocol, VUs represent the 10-times quotient of the samples’ optical density (OD) and the OD of a kit-provided cut-off control. VU > 11 is considered positive, while VU < 9 is determined negative. VU values between 9 and 11 are considered equivocal/weakly positive.

CSF samples of the control group were additionally tested by mass spectrometry to identify herpesvirus-specific peptides. HSV-1 peptides were searched for from 73 HSV-1 proteins from the UniProt database. In addition, peptides were searched for from 75 HSV-2 proteins. In the same search, peptides from humans, bacteria, and fungi were present; however, no peptides for HSV-1, HSV-2, or other viruses were detected.

In selected cases, when both serum and CSF were negative for HSV-1-specific IgG against HSV-1, serum samples were tested for serology against *human herpesvirus 6* (HHV-6)-specific IgG/IgM class antibodies was tested (ELISA-VIDITEST anti-HHV-6 IgG/IgM, Virotech Diagnostics GmbH, Dietzenbach, Germany, virotechdiagnostics.com, accessed on 7 July 2024). The results are given in Table S4.

## 3. Results

### 3.1. Transelectron Microscopy (TEM) Reveals Intracellular Herpesvirus Particles in CSF

**Figure 1:**
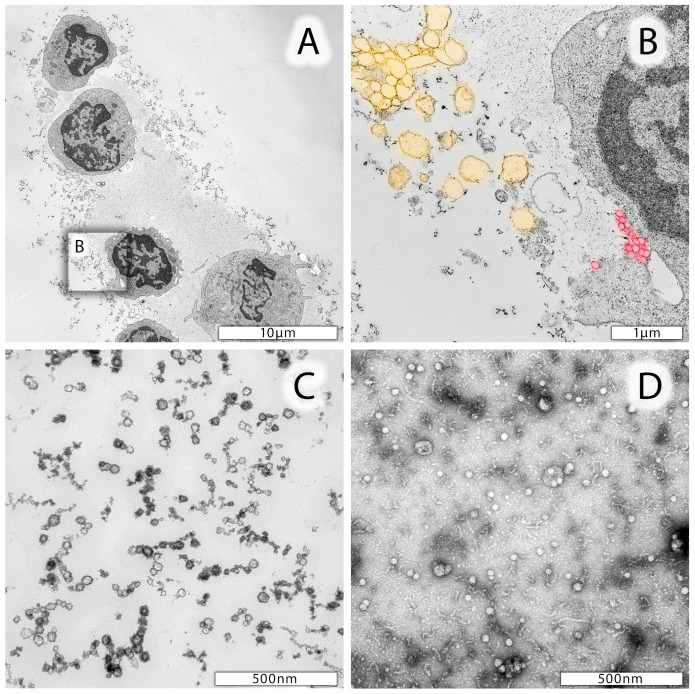
Representative electron micrographs of leukocytes from CSF. (**A**) Lymphocytes and one monocyte in the lower part of the image. Cells are surrounded by a protein matrix and numerous vesicles differing in size. (**B**) Enlarged area of a lymphocyte marked in (**A**) with likely extruding microparticles highlighted in yellow and exosomes highlighted in red. (**C**) Smaller (<40 nm in diameter) particles in CSF specimen which may be due to proteins, oxidized phospholipids, and nucleic acids. (**D**) Negative staining preparation of mesenchymal stem-cell-derived exosomes.

Cellular elements of CSF specimens were examined by TEM. As exemplified in Figure 1, the CSF contained lymphocytes at various activation states, dendritic cells, few granulocytes, fine-structured protein nets, and phospholipid vesicles (Figure 1A). In addition, extracellular vesicles (EVs) with variable diameters in size and electron densities were identified (Figure 1B,C). EVs, including exosomes, were found adjacent to the cell surface of lymphocytes or monocytes, documenting active EV release (Figure 1A and enlarged insert Figure 1B). Exosomes’ sizes range between 50 and 120 nm in diameter (Figure 1B, highlighted in red), whereas microparticles are larger (200–500 nm in diameter, Figure 1B, highlighted in yellow). Size determinations also led to the identification of very small particles (<40 nm in diameter), likely to represent oxidized phospholipids (Figure 1C). In addition, stem-cell-derived exosomes (XoGlo^R^, Kimera Labs Inc., Miramar, FL, USA, Kimeralabs.com, accessed on 8 July 2024) were processed for negative staining, using a 1:10-diluted stem cell exosome preparation and 1% uranyl acetate. Negative staining of stem-cell-derived exosomes’ morphological properties is shown in Figure 1D.

**Figure 2:**
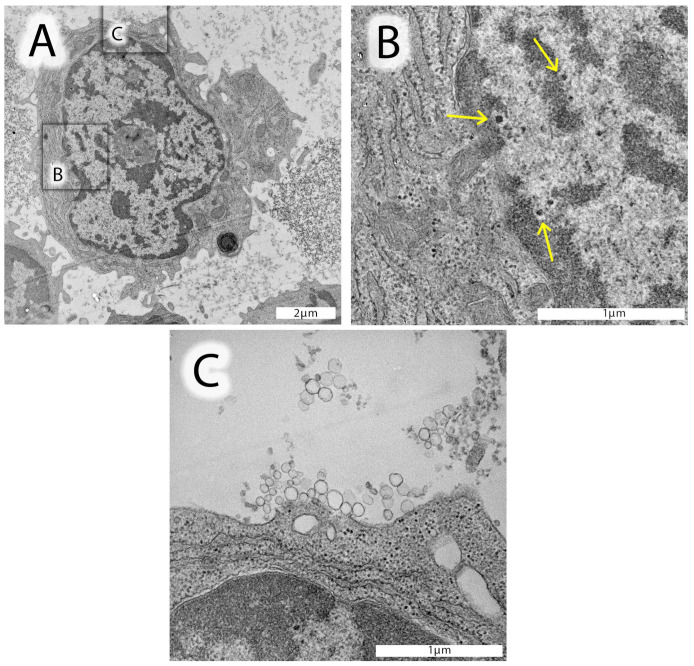
Electron micrographs of leukocytes from a selected AF patient #15159. (**A**) Lymphocyte from the CSF with a well-structured, metabolically active nucleus, mitochondria, and endoplasmic reticulum. The nucleus of this lymphocyte contains homo- and heterochromatin and a well-identified nucleolus in the center. Distinct, highly electron-dense spots, structurally similar to herpesvirus nucleocapsids devoid of an envelope, can be identified in the distal area of the heterochromatin. (**B**) Enlarged insert of the nuclear area (black square in (**A**)) shows these likely herpesvirus nucleocapsids with a characteristically light halo surrounding the nucleocapsid (yellow arrows). (**C**) Enlarged insert area from (**A**) documenting the presence of exosomes (50–120 nm in diameter) close to lymphocyte’s cell surface. In the upper part of this image insert, very small vesicles can be identified, likely corresponding to proteins, oxidized phospholipids, and/or nucleic acid aggregates.

The ultrastructure of nucleus-containing leukocytes from one of our AF patients (patient #15159) is shown in Figure 2. In a number of lymphocytes examined, well-identified, distinct black-spotted fragments were detected, which may represent incomplete herpesvirus particles (Figure 2A, and enlarged inserts in Figure 2B,C). These distinct black fragments were also identified in other AF patients’ leukocytes from CSF (Figure S4A,B). Moreover, these lymphocytes were also surrounded by numerous exosomes, likely released from the adjacent cell (Figure 2A, and enlarged insert Figure 2C). Figure 2C also shows even smaller particles similar to those in Figure 1B,C, which are likely due to phospholipids, proteins, and nucleic acid-bearing aggregates.

### 3.2. Differential Centrifugation to Enrich Exosomes from CSF

**Figure 3:**
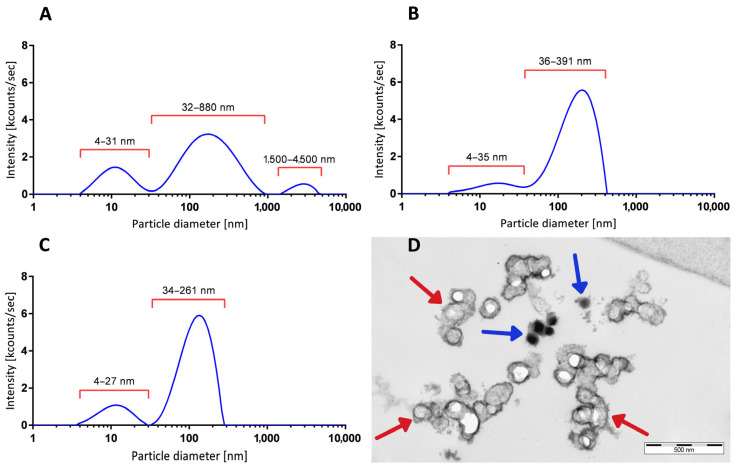
Vesicle size distributions from CSF-derived particles (EVs). (**A**) DLS-generated size distribution profile of cell-free CSF after step 1 centrifugation from a representative AF patient, with peaks at 12.20 nm, 215.00 nm, and 2790 nm in diameter. (**B**) Size distribution profile of another patient’s preparation after step 3 ultracentrifugation. Enriched EVs show a major peak at 183.5 nm in diameter and a smaller peak fraction with 16.0 nm in diameter. (**C**) DLS-generated size distribution pattern of highly purified mesenchymal stem-cell-derived exosomes (XoGlo^R^, Kimeralabs.com) showing a major peak at 124.0 nm and a smaller peak at 11.90 nm in diameter. For vesicle size distribution plots, the relative intensities (kcounts/s, y-axis) were plotted against the log_10_ values of particles’ diameters (x-axis). (**D**) Ultrastructure of cryopressure-processed, highly purified mesenchymal stem-cell-derived exosomes (XoGlo^R^, Kimeralabs.com). Preparation contains lipid-bilayer-enclosed vesicles (red arrows) and lipid-bilayer-negative electron-dense aggregates (blue arrows).

Differential centrifugation was applied to enrich exosomes from CSF specimens. The first centrifugation removed leukocytes (Figure S2, step 1). Figure 3A shows a representative vesicle size distribution of cell-free CSF (patient #15129) determined by dynamic light scattering (DLS). The smallest peak likely represents proteins, phospholipids, and/or nucleic acid particles, which range 4–31 nm in diameter (Figure 3A, see also Figure 1C). The peak with the highest intensity represents particles ranging from 30 to 880 nm, and only a small particle fraction was 1.5–4.5 µm in size. These larger particles were most probably fragmented mitochondria and apoptotic bodies (also confirmed by electron microscopy and flow cytometry using staining with mitotracker and propidium iodide). Figure 3B shows a representative size distribution profile of CSF exosomes enriched by ultracentrifugation (Figure S2, step 3). The peak with highest intensity ranged between 36 and 391 nm, corresponding to exosomes (Figure 1B and Figure 2C). Highly purified mesenchymal stem-cell-derived exosomes (XoGloR, Kimeralabs.com) gave similar DLS results (Figure 3C). Figure 3C shows two peaks by DLS, one between 34 and 261 nm, and one of 4–27 nm. Electron micrographs of mesenchymal stem-cell-derived exosomes prove the presence of exosomes and smaller particles which may be protein aggregates (Figure 3C,D).

### 3.3. CSF-Derived Exosomes Contain Virus-Encoded and Host-Derived Inflammatory miRNAs

CSF-derived exosomes from controls and patients with SAH, AF, SZ, and mixed neuroinflammatory diseases (Table 1) were subjected to RNA isolation and RT-qPCR-based detection of HSV-1- and host-derived miRNAs.

**Figure 4:**
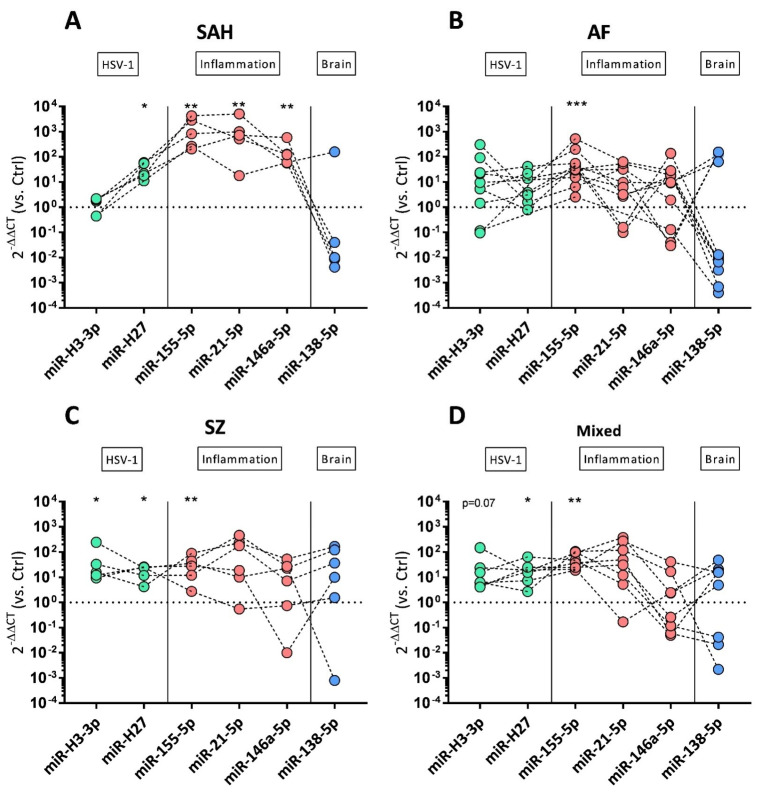
miRNA expression profiles of CSF-derived exosomes’ preparations. Log_10_ fold changes of HSV-1-derived miR-H3-3p and miR-H27 (green symbols), cellular inflammatory miR-155-5p, miR-21-5p, miR-146a-5p (red symbols), and brain-derived miR-138-5p (blue symbols) are shown as dot plots in SAH (**A**), AF (**B**), SZ (**C**), and non-traumatic/non-psychiatric patients (**D**). Log_10_ fold changes were calculated using the 2^−ΔΔCT^ method. Control (Ctrl)-derived exosomal preparations served as reference, and the dotted horizontal line (fold change = 1) represents the cut-off for elevated transcripts. For viral miR-H3-3p, *n* = 5 SAH, *n* = 10 AF, *n* = 5 SZ, and *n* = 7 non-traumatic/non-psychiatric patients were analyzed and normalized against *n* = 3 Ctrl samples. For viral miR-H27, *n* = 5 SAH, *n* = 7 AF, *n* = 5 SZ, and *n* = 7 non-traumatic/non-psychiatric patients were analyzed and normalized against *n* = 4 Ctrl samples. For host miRNAs, *n* = 5 SAH, *n* = 11 AF, *n* = 6 SZ, and *n* = 8 non-traumatic/non-psychiatric patients were analyzed and normalized against *n* = 5 Ctrl samples. Wilcoxon rank-sum test was performed to analyze differences against controls (Ctrl). Significant differences are marked by * *p* ≤ 0.05, ** *p* ≤ 0.01, and *** *p* ≤ 0.001.

Table S5 summarizes the numbers of positively detected miRNA species in exosomal preparations of all patients. MiR-H3-3p, miR-H6-3p, and miR-H27 were detected in all tested specimens. Expression intensities varied over a broad range, but miR-H6-3p, encoded outside of LAT, was found to be the most stably expressed miRNA and thus served as a normalization control for quantification of viral miR-H3-3p and miR-H27 (Table S6, Figure S3). Residual viral miR-H2-3p, miR-H4-3p, and miR-H4-5p were negative in a number of samples. In the control samples, viral miR-H4-3p and miR-H4-5p were detected in all preparations, whereas miR-H2-3p was completely absent in controls (Ctrl, Table S5). Host-derived miR-155-5p, miR-21-5p, and miR-146a-5p were quantified, and neuronal miR-138-5p was included to prove exosomes’ originating from neuronal cells. Figure 4 summarizes the miRNA expression profiles for patients with SAH, AF, SZ, and mixed neuroinflammatory diseases. miRNA expression was calculated as fold changes (2^−ΔΔCT^) against controls as the reference group.

SAH patients were unique in presenting very low levels of viral miR-H3-3p (Figure 4A). The highest miR-H3-3p levels were detected in individual AF patients (Figure 4B); however, miR-H3-3p was significantly elevated in patients with SZ (Figure 4C). Viral miR-H27 was elevated in all groups except AF (Figure 4B).

Inflammatory miR-155-5p was significantly elevated in all groups, and the highest levels were found in SAH and AF patients (Figure 4A,B). Expression of miR-155-5p further correlated with miR-H27 expression (Spearman r = 0.44, *p* = 0.02; Figure S5A). Enhanced expression of inflammatory miR-21-5p and miR-146a-5p was restricted to SAH patients (Figure 4A). In contrast to the other groups, none of the SAH preparations had decreased miR-146a-5p levels (Figure 4A). For individual SAH and psychiatric patients, multiple samples were tested (Figure S6). Inflammatory miRNAs were highly expressed yet unchanged in SAH (Figure S6A), but they were found to increase in psychiatric patients when testing follow-up samples over a longer period of time (Figure S6B). Neuronal miR-138-5p was enriched in individual samples of each group, but low expression levels were detected in 4/5 SAH and 7/11 AF patients (Figure 4A,B). Remarkably, the only SAH patient sample with positive miR-138-5p originated from a case without an aneurysm (patient #18858, Table S2). The low expression levels of miR-138-5p appear to correlate with low expression levels of miR-H3-3p. This is best noticed in SAH-derived specimens. Accordingly, miR-138-5p and miR-H3-3p showed a significant correlation (Spearman r = 0.46, *p* = 0.01, Figure S5B).

### 3.4. Herpesvirus Antibodies in Plasma, Serum and CSF

HSV-1-directed IgG titers were determined in serum or plasma and the corresponding CSF specimen. An overview of positively detected samples is provided in Table S3. Accordingly, 4/4 SAH patient specimens were positive for HSV-1 IgG in serum, and 2/3 in CSF. In both positive CSF specimens from SAH patients, HSV-1 titers were higher in CSF when compared to plasma. In the AF group, 7/10 plasma/serum samples were HSV-1 IgG-positive, and 3/8 CSF samples (including one weakly positive result). HSV-1 titers against HSV-1 were always higher in plasma when compared to CSF. In SZ patients, 1/3 plasma/serum samples and 3/6 CSF specimens were positive for HSV-1. Specimens from the mixed neuroinflammatory group of patients were mostly positive for HSV-1 IgG (3/4 plasma samples and 4/5 CSF samples). The CSF sample of patient T#1 with VZV meningitis exhibited higher IgG titers in CSF when compared to plasma, but similar to SZ with low titers. For the control group, no serum was available, and only one out of five CSF specimens was detected as positive. CSF samples from the control group were additionally examined using mass spectrometry to detect peptides associated with HSV-1/-2. However, none of the HSV-1 peptides from a pool of 73 HSV-1 variants nor any of the 75 HSV-2 proteins listed in the UniProt database were identified in the CSF samples. This implies that these samples were negative for HSV-1/2. HHV-6-directed antibodies were detected in one SZ patient (#16362).

### 3.5. Neuronal Cell Damage and Oxidative Stress Linked to Exosomal miRNAs

**Figure 5:**
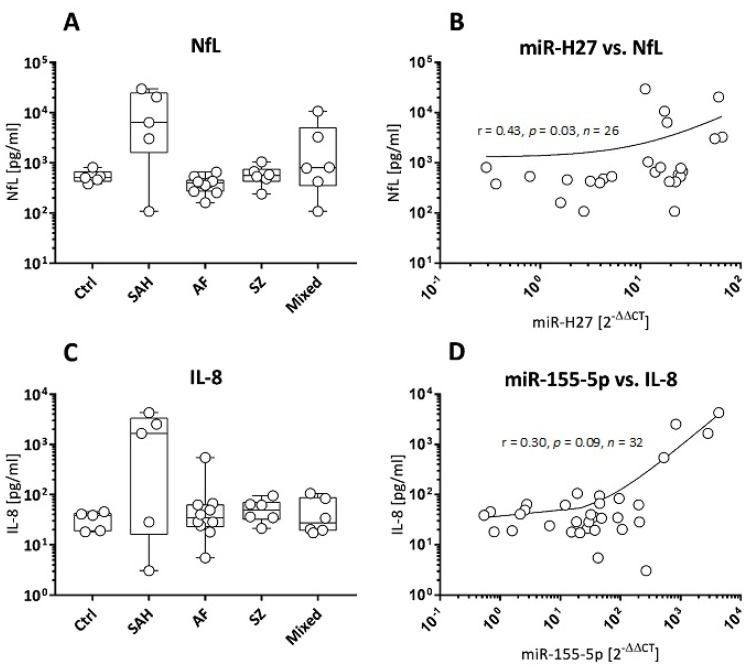
NfL and IL-8 concentrations in correlation to viral and host miRNAs. (**A**) CSF NfL concentrations (pg/mL), presented as box plots (median + range), for *n* = 5 controls (Ctrl) and patients with SAH (*n* = 5), AF (*n* = 8), SZ (*n* = 6), and mixed neuroinflammatory diseases (*n* = 6). (**B**) Correlation between *n* = 26 NfL concentrations (**A**) and corresponding viral miR-H27 fold changes. (**C**) CSF IL-8 concentrations (pg/mL), presented as box plots (median + range), for *n* = 5 controls (Ctrl), and patients with SAH (*n* = 5), AF (*n* = 10), SZ (*n* = 6), and mixed neuroinflammatory diseases (*n* = 6). (**D**) Correlation between *n* = 32 IL-8 concentrations (**C**) and corresponding host-derived miR-155-5p fold changes. Correlations are presented as scatter plots; r: Spearman correlation coefficient, *p*: significance, *n*: number of specimens.

Neurofilament light chain (NfL), an axonal damage marker, and the chemokine and oxidative stress-related IL-8 were tested to find out whether miRNA expression patterns would correspond to cell stressors such as tissue damage and oxidative stress. NfL turned out to be highest in CSF of 4/5 SAH patients (3044–29,760 pg/mL, Figure 5A), and lowest NfL concentrations were found in CSF from AF (median: 389.3 pg/mL) and SZ patients (median: 559.5 pg/mL). The latter concentrations were similar to controls (median: 508 pg/mL, Figure 5A). Elevated NfL was further detected in 2/5 patients with non-traumatic/non-psychiatric diseases (3295–10,734 pg/mL, Figure 5A). When correlating NfL concentrations with HSV-1-derived miRNA expressions, miR-H27 turned out to be linked to NfL (Spearman r *=* 0.43, *p =* 0.03, Figure 5B), but miR-H3-3p did not (Spearman *r* = 0.01, *p* = 0.98). With a single exception, the highest NfL concentrations (>600 pg/mL) were restricted to miR-H4-3p-positive samples (Figure S5D). Table S8 presents an overview of all correlations between NfL and the miRNAs displayed in Figure 4.

Figure 5C shows that the oxidative stress marker and chemokine IL-8 was elevated in individual specimens of all groups. The highest IL-8 levels were detected in 3/5 SAH and 1/10 AF patients (1670–4313 pg/mL, 548 pg/mL, respectively, Figure 5C). IL-8 concentrations further showed a moderate correlation with fold changes of host-derived miR-155-5p (Spearman r = 0.30, *p* = 0.09, Figure 5D). Table S8 presents an overview of all correlations between IL-8 and the miRNAs displayed in Figure 4.

## 4. Discussion

### 4.1. Encephalitis in the Context of Viral Infections and Immunity

Encephalitis-related diseases have long been linked to viral infections, including herpesviruses [1]. Herpesviruses cause lifelong latent infections with limited viral gene expression and no severe disease manifestation. Triggered by a variety of stressors (tissue damage, mental and social stressors, chronic inflammation, other virus reactivations), immune control weakens, and herpesviruses may reactivate and switch to productive (lytic) replication [8,42,43,44]. Reactivation can be followed by acute disease and severe morbidities such as herpes simplex encephalitis (HSE) [8,45]. Secondary immune responses in HSE may proceed into autoimmune conditions, in that autoantibodies against NMDAR are also found in SZ [3,46]. In psychiatric diseases with low-grade inflammation in the brain, antibodies against HSV-1 are indeed elevated in AF, SZ, and bipolar disorder patients [5,6,47,48]. Virus latencies such as HSV-1 cannot be regarded as monocausal factors for inflammation, but anti-apoptotic [49,50,51] and immune-modulatory functions exerted by HSV-1-encoded LATs may result in exhausted CD8^+^ T cells [51,52] and inflammation with TNF-α or interferon-γ induction by CD3^+^/CD8^+^ T cells and CD68^+^ macrophages in trigeminal ganglia [53]. These observations imply that latency itself is linked to inflammation, yet in the absence of neuronal cell death. Increased LAT copy numbers also correlate with decreased Beclin-1 and BDNF (brain-derived neurotrophic factor) expression, resulting in increased oxidative stress and decreased autophagy [54]. Viral miRNAs are the main LAT-encoded transcripts [18]. Thus, viral miRNAs constitute promising biomarkers in the context of neuronal HSV-1 infection, recurrent reactivation, and neuroinflammation in patients encountering various stress conditions. The aim of this study was to determine whether HSV-1-derived miRNAs can be detected in CSF-derived exosomes in patients with psychiatric disorders and whether exosomal miRNA profiles differ from other neuroinflammatory conditions. Protein biomarkers such as axonal damage marker NfL and oxidative stress marker IL-8 were quantified to determine neuronal damage and oxidative stress [35,55].

### 4.2. Cells and Exosomes in CSF from Psychiatric Patients

Cells and extracellular vesicles, including exosomes, were studied in an investigation of their ultrastructural properties in CSF of patients with neuroinflammation and a psychiatric phenotype. Ultrastructural investigations of leukocytes in the CSF identified incomplete nucleocapsids within the nucleus, and enveloped herpesvirus-like particles were eventually found in the cytoplasm (Figure S4B). Black-spotted fragments likely document herpesvirus particles. Due to its fast and unbiased approach, TEM is widely used in viral diagnostics for a variety of specimens including CSF [56]. In CSF, TEM identified herpesviruses as the most abundant virus family in nearly 500 cases between 2004 and 2008 [56]. Viral identification by TEM is based on morphological properties [57]. Accordingly, our results correlated morphologically and size-wise with previous studies describing cell-enclosed herpesviruses by TEM, including the characteristic light halo around the nucleocapsid (Figure S4B) [56,58].

TEM further showed that the cells with virus-derived nucleocapsids in the nucleus were surrounded by a huge amount of extracellular vesicles (including exosomes), suggesting active vesicle and exosome release. These vesicles were characterized by morphological criteria and DLS measurements to confirm their exosomal properties [13], using highly purified mesenchymal stem-cell-derived exosomes as quality control. DLS-based vesicle size determinations are dependent on vesicle concentrations of the analyte, influencing the measured size [59]. In our study, CSF material was often limited, and exosomal properties must be confirmed in future studies by zeta potential or exosomal protein markers such as CD63 or CD81 [13,59].

### 4.3. Exosomal miRNAs to Delineate HSV-1 Replication and Neuroinflammation

Exosomes are highly enriched in miRNAs [17,22], which makes them advantageous for identifying biomarkers and transcripts of host- and pathogen-derived origin. Based on the successful identification of HSV-1-encoded miRNAs in CSF exosomes of HSE patients [60], we here determined viral miRNAs in CSF exosomes of patients with AF, SZ, SAH, and a variety of other (mixed) neuroinflammatory diseases. HSV-1-derived miR-H3-3p, miR-H6-3p, and miR-H27 were detected in all tested samples, and miR-H6-3p was found to be most stably expressed. MiR-H6-3p promotes latency by targeting viral ICP4, an immediate early protein in replication [18]. In contrast to the other viral miRNAs in this study, miR-H6-3p is encoded outside the viral LAT [18], which supports its role as a normalization factor for LAT-derived viral miRNA expression analysis.

Viral miR-H3-3p, which shows sequence complementary to ICP34.5, a major viral factor involved in HSV-1 latency [18,61], was low in SAH preparations but was enriched in many AF samples, all SZ samples, and samples from patients with various other neuroinflammatory conditions. MiR-H3-3p inhibits the expression of SMAD4, a transcription factor of the TGF-β pathway, which is downmodulated by HSV-1 infection [62]. In contrast to miR-H3-3p, miR-H27 was significantly elevated in SAH. MiR-H27 is linked to active viral replication and inhibits the transcription expression of KLHL24, a host repressor of viral replication [21]. As a consequence, miR-H27 should be absent in latency, should increase during reactivation, and should be highly elevated in states of lytic infection [63]. The role of miR-H27 as a strong indicator of more frequent productive replication is supported by a significant correlation with host-derived miR-155-5p. The highest levels of the latter were detected in SAH patients and some AF patients. Thus, miR-155-5p upregulation indicates regulatory events in the context of viral infections in both trauma-associated neuroinflammation and psychiatric diseases [64,65,66].

Since miR-21-5p and miR-146a-5p were also elevated in exosomal CSF preparations of SAH patients, tissue damage responses due to leukocyte infiltration [67] are more likely than brain-derived stressors, which we followed by increased miR-138-5p. Low neuronal miR-138-5p was found in 4/5 SAH patients. The miR-138-5p-positive SAH specimen was derived from a patient who (unlike all other SAH patients) did not suffer from an aneurysm (patient #18858, Table S2). Thus, low miR-138-5p appears to correlate with increased leukocyte infiltration into the brain, which indeed is a possible consequence of cerebral aneurysms. MiR-138-5p targets and represses ICP0, the viral transcription factor that needs to be silenced during HSV-1 latency [21,31]. It is noteworthy that detection levels of miR-138-5p and the presence of IgG against HSV-1 in CSF resulted in a weak but not significant negative correlation (Spearman r = −0.36, *p* = 0.06, Figure S5C). This may suggest that higher levels of miR-138-5p could potentially indicate a lower degree of immune activation.

### 4.4. Tissue Damage Separates SAH from Neurological Disorders

As described earlier, HSV-1 reactivation may be triggered by trauma, psychiatric stressors, and cell damage and may result in increased neuronal damage, linked to miR-155-5p [6,13,68]. Indeed, higher levels of NfL appear to be restricted to trauma-associated inflammation and correlated with viral miR-H27, indicative of viral replication, but not with latency-associated miR-H3-3p. This is supported by a correlation of miR-H27 and miR-155-5p, linking neuroinflammation to viral replication. However, miR-H27 did not correlate with IL-8, whereas miR-155-5p presented a positive yet not significant correlation. This indicates IL-8 to be less of a marker for HSV-1 infection than for neuroinflammation in general, as supported by our previous results [35].

In summary, the current analysis provides evidence for the regulatory role of HSV-1-encoded miRNAs targeting the neurological virulence factor ICP34.5 in neuroinflammation, but not in trauma-induced tissue damage such as SAH. HSV-1-encoded miR-H27 is valid to substantiate the frequencies of HSV-1 reactivation, especially when compared with cellular miR-138-5p targeting ICP0, the latter related to virus silencing. Eukaryotic miR-155-5p plays a unique role by suppressing the interferon response of the host and simultaneously activating HSV-1 gene expression.

### 4.5. Limitations and Strengths of the Current Study

A major limitation of the current study is the low sample number in each patient group, alongside limited material, which further prevented several markers from being determined for each sample. Although psychiatric diseases can be distinguished by clinical phenotypes and can be assigned to subgroups, high individuality remains. As explained in a recent review by [69], a great part of this heterogeneity may occur by oxidative stressors, often leading to redox imbalances. The authors explain pathways regulated by transcription factors, calcium signaling, iron toxicities, and the supply of highly relevant antioxidants such as glutathione to maintain synaptic functions and neurotransmitter supply [69]. In addition, virus infections constitute major modifiers of disease phenotypes, especially in schizophrenia [70]. The preliminary findings of this study must therefore be consolidated by larger cohorts to draw reliable and statistically significant conclusions. In order to provide additional evidence for the valuable insights gained from studying both HSV-1-encoded miRNAs and cellular miRNA species in relation to the dynamics of latency and reactivation of HSV-1, this analysis could complement a recent study that identified exosomal miR-H2-3p and miR-H4-3p as potential CSF biomarkers for HSE [60]. These two miRNAs are most abundant in latently infected human trigeminal ganglia [71] but were widely undetected in our study. Individual patients presented negative in HSV-1 serology despite the positive detection of HSV-1-encoded miRNAs. The selective role of neuronal miR-138-5p and low or even negative HSV-1 antibody titers impacts further investigations. This observation indicates that HSV-1 reactivation may occur in the absence of humoral immune competence which might further emphasize the role of miR-155-5p on immunity. In addition to the HSV-1-encoded miRNAs studied here, other herpesviruses’ miRNAs are highly relevant to be analyzed in future studies [72]. The recently identified HHV-6A-encoded miR-aU14 has been shown to interfere with cellular type I interferon responsiveness [73]. Previous studies further report the involvement of Borna Disease Virus in patients with neuropsychiatric disorders [74], highlighting the impact of neurotropic viruses in neuroinflammation. Additional TEM analysis is also favorable, since a proper identification of virions may be more indicative compared to viral transcripts [75].

To our knowledge, this is the first study addressing HSV-1-derived miRNAs in CSF exosomes of patients with psychiatric and trauma-associated encephalitis and comparison with inflammation-negative CSF. This approach bears reasonable potential for extended studies and higher numbers of patient samples to be included.

## 5. Conclusions

To answer the question of whether active/reactivated and/or latent HSV-1 infection would play a role in neuroinflammation, HSV-1-encoded miRNAs were tested for their abundance in CSF exosomes derived from patients with SAH, AF, SZ, and mixed neuroinflammatory diseases. In selected CSF samples, TEM identified leukocyte-enclosed nucleocapsid structures that could be derived from herpesvirus particles. These leukocytes also showed active release of vesicles including exosomes. In addition, elevated exosomal miR-H27 in nearly all specimens supports the contribution of replicating HSV-1. Different from miR-H27, elevated viral miR-H3-3p expression was restricted to SZ-derived specimen. All patient-derived exosomal RNA preparations were positive for miR-155-5p, confirming a neuroinflammatory phenotype. Low exosomal miR-138-5p in SAH and AF indicates that exosomes might originate from brain-infiltrating leukocytes. Different from psychiatric patients, inflammation in SAH patients results from tissue damage, which may also reactivate HSV-1, and this process is supported by elevated NfL in the CSF of SAH patients.

So far, the sample numbers for every patient group are comparatively small. Thus, the selective value of HSV-1-encoded miRNA species tested here needs to be tested in larger patient cohorts, and CSF-derived exosomes from HSE patients should be included to confirm the replication-linked miR-H27.

## Figures and Tables

**Table 1:** Characteristics of patient groups and controls. * Detailed demographic and clinical data are summarized in Table S2. ** Less-well-defined neuroinflammatory diseases were from *n* = 1 patient HLH in remission (age 1), from *n* = 1 patient with hydrocephalus (NPH, age 65), *n* = 1 patient with varicella zoster virus (VZV) meningitis (age 74), *n* = 2 patients with facial palsy (age 49 and 66), *n* = 1 patient with newly diagnosed multiple sclerosis (MS, age 15), *n* = 1 patient with dissociative disorder (age 29), and *n* = 1 patient with major hemorrhage following surgical resection for Glioblastoma °IV (age 63).

Group	ICD-10 Class	Total	Male	Female	Mean Age (Range)
Non-neuroinflammatory controls * (Ctrl)	n. a. *	*n* = 5	*n* = 2	*n* = 3	60 (53–70)
Subarachnoid hemorrhage * (SAH)	I60	*n* = 5	*n* = 3	*n* = 2	52 (40–68)
Affective spectrum disorder (AF)	F30-F33	*n* = 11	*n* = 5	*n* = 6	40 (28–57)
Schizophrenic spectrum disorder (SZ)	F20-F25	*n* = 6	*n* = 3	*n* = 3	47 (23–64)
Other neuroinflammatory diseases *,**	Multiple diagnoses *	*n* = 9	*n* = 3	*n* = 6	39 (1–75)

## Data Availability

Data are contained within the article and Supplementary Materials.

## Supplementary Materials

The following supporting information can be downloaded at https://www.mdpi.com/article/10.3390/cells13141208/s1: Figure S1: Selected HSV-1-derived miRNAs and their location within the HSV-1 genome; Figure S2: Overview on experimental procedure as described in Section 2; Figure S3: Viral miRNA stability; Figure S4: TEM images from CSF cell preparations of two AF patients; Figure S5: Host and HSV-1-derived miRNA expression; Figure S6: miRNA profile in CSF-derived exosomes for follow-up samples; Table S1: Selected targets of viral- (*hsv1*-) and host-derived (*hsa*-) miRNAs in the context of HSV-1 infection; Table S2: Additional clinical data on non-neuroinflammatory controls (Ctrl) and patients with SAH and mixed neuroinflammatory diseases (Mixed); Table S3: HSV-1/-2 serology in serum and CSF; Table S4: HHV-6 serum serology; Table S5: Positive detected samples for each viral miRNA in each group; Table S6: Candidate housekeeping gene comparisons; Table S7: CT values for viral miRNAs in CSF-derived exosomes; Table S8: Correlation of host- and HSV-1-derived miRNAs with NfL and IL-8. References [76,77,78,79,80,81,82,83] are cited in the Supplementary Materials.
