# Supplementary material for: HSV-1 and Cellular miRNAs in CSF-Derived Exosomes as Diagnostically Relevant Biomarkers for Neuroinflammation
Source: Cells. 2024 Jul 17;13(14):1208. doi: 10.3390/cells13141208 (PMC11275151; doi:10.3390/cells13141208)
Supplement: Supplementary file 1 [file cells-13-01208-s001.zip › cells-3036537-supplementary.pdf]

MDPI cells

Supplement

Table S1. Selected targets of viral- (*hsv1*-) and host-derived (*hsa*-) miRNAs in the context of HSV-1 infection.

MiRNA	Target	Target function	Reference(s)
hsv1-miR-H2-3p	ICP0	Viral transcription activator	[18]
	DDX41	Host RNA helicase	[76]
	SMAD3	Host mediator of TGF-β signaling	[62]
	SMAD4	Host mediator of TGF-β signaling	[62]
hsv1-miR-H3-3p	ICP34.5	Viral neurovirulence factor, inactivating autophagy	[18]
	SMAD4	Host mediator of TGF-β signaling	[62]
hsv1-miR-H4-3p	ICP34.5	Viral neurovirulence factor, inactivating autophagy	[18,77]
hsv1-miR-H4-5p	CDKN2A	Host suppressor of PI3K/AKT pathway	[63,78]
	SMAD3	Host mediator of TGF-β signaling	[62]
hsv1-miR-H6-3p	ICP4	Viral transactivator of viral genes in active infection	[18]
hsv1-miR-H27	KLHL24	Host repressor of viral gene replication	[21]
hsa-miR-21-5p	PDCD4	Host regulator of apoptosis	[79]
	PD-1	Host regulator of cell death	[80]
hsa-miR-138-5p	ICP0	Viral transcription activator	[31,32]
hsa-miR-146a-5p	IRAK1	Host modulator of NF-κB activation	[81]
hsa-miR-155-5p	SOCS-1	Host suppressor of JAK-STAT pathway	[65,82]
	SRSF2	Host transcriptional activator of HSV-1 gene expression	[66]

Table S2. Additional clinical data on non-neuroinflammatory controls (**Ctrl**), patients with SAH, and various other neuroinflammatory diseases (#14689, #17345, #17442, T#1, T#3, T#4, T#5, T#6,). SAH patients were classified according the grading system established by Hunt and Hess [83]; ALL: acute lymphoblastic leukemia; Ctrl: non-neuroinflammatory controls; HLH: hemophagocytic lymphohistiocytosis; MS: multiple sclerosis; NPH: normal pressure hydrocephalus; SAH: Subarachnoid hemorrhage VZV: varicella zoster virus.

Pat. ID.	Diagnosis	Age	Gender	Further clinical information
TK1	Ctrl	62	M	M19.0, M75.2, R41.8
TK2	Ctrl	53	F	D48.0, M79.9, N10, O32.1, O47.9, O80.1, R11, R29.8, R50.9, S01.2, S02.20, S02.21, S62.5, V14.2, W01, Y94.8, Y96.0, Z00.00 (V700)
TK3	Ctrl	58	F	I87.2, J30.10, L20.9, L28.0, L50.8, M21.5, M99.5, R29.8, R42, R49.8, S81.0, S93.4, T91.8, W01, Y94.8, Y96.9, Z01.5
TK4	Ctrl	70	M	E11.8, E78.5, H81.1, H90.3, H90.7, I10, K11.2, M35.3, N40, R07.4, R86.1, R68.7, T99.99
TK5	Ctrl	56	F	A04.7, D86.1, D86.2, E06.9, G47.3, H04.10, H04.5, I10, K07.61, K80.2, K80.8, M13.9, M25.5, M79.1, N80.5, N80.9, R10.2, R10.3, R10.4, R41.8, R52.2, R91, T81.0, Y83, Z34.00 (V220), Z71.2
#14690	SAH	46	F	SAH, Hunt and Hess Grade II; aneursyms (middle cerebral artery, and anterior cerebral artery); hydrocephalus malresorptivus; secondary diagnoses: Type 1 diabetes, hypothyroidism, hypertension; CSF sampling on day 1 after initial bleeding
#18858	SAH	40	M	SAH, Hunt and Hess Grade II; no aneursym; secondary diagnoses: hypertension; CSF sampling on day 4 after initial bleeding
#19365	SAH	68	F	SAH and ICB (intracerebral bleeding), Hunt and Hess Grade III; aneursym (anterior cerebral artery); vasospasm, urinary tract infection; secondary diagnoses: Hypothyroidism, hypertension; CSF sampling on day 8 after initial bleeding
#19384	SAH	45	M	SAH, Hunt and Hess Grade II; aneursym (vertebral artery); hydrocephalus malresorptivus; CSF sampling on day 4 after initial bleeding
#19442	SAH	61	M	SAH, Hunt and Hess Grade III; aneursym (anterior cerebral artery); secondary diagnoses: Type 2 diabetes, alcohol abusus; CSF sampling on day 4 after initial bleeding
T#1	VZV meningitis	74	M	G02.0
T#3	Dissociative disorder	29	M	F41.0
T#4	MS	15	F	G35.0 (primary manifestaion of multiple sclerosis, treatment naive)
T#5	VZV-associated facial palsy	66	F	G51, B02.8
T#6	Facial palsy	49	F	G51.1
#14689	NPH	65	F	G91
#17345	HLH	1	F	D76.1 (ALL and HLH at clinical remission)
#17442	Surgical hemorrhage	63	M	C71.9 (Glioblastoma °IV)

Table S3. HSV-1/-2 serology in serum and CSF. The IgG positivity against HSV-1/-2 is given for CSF and corresponding serum samples. Serological testing was performed as described in Section 2; Results are given as Virotech Units (VU). According the manufacturer's protocol, VU represent the 10-times quotient of the samples' optical densitiy (OD) and the OD of a kit-provided cut-off control. VU>11 are considered positive, while VU<9 are determined negative. VU between 9-11 are considered equivocal/weakly positive. Plasma of *n* = 4 SAH, *n* = 10 AF, *n* = 3 SZ, and *n* = 4 patients with mixed inflammatory diseases was analyzed, while no plasma was available from Ctrl samples. CSF of *n* = 5 Ctrl, *n* = 3 SAH, *n* = 8 AF, *n* = 6 SZ, and *n* = 5 patients with mixed inflammatory diseases was analyzed; (n. d. = not determined); AF: Affective spectrum disorder; Ctrl: non-neuroinflammatory controls; HLH: hemophagocytic lymphohistiocytosis; SAH: Subarachnoid hemorrhage; SZ: Schizophrenic spectrum disorder; VZV: varicella zoster virus; *VU: Virotech units; IgG classification: VU<9: negative; VU=9-11: equivocal/weakly positive; VU>11: positive.

Pat. ID.	Diagnosis	IgG plasma [VU]*	IgG plasma classification*	IgG CSF [VU]*	IgG CSF classification*
TK1	Ctrl	n. d.	n. d.	8.9	Negative
TK2	Ctrl	n. d.	n. d.	7	Negative
TK3	Ctrl	n. d.	n. d.	1	Negative
TK4	Ctrl	n. d.	n. d.	13	Positive
TK5	Ctrl	n. d.	n. d.	5	Negative
#19442	SAH	13	Positive	17	Positive
#19384	SAH	14	Positive	18	Positive
#18858	SAH	16	Positive	0	Negative
#14690	SAH	n. d.	n. d.	n. d.	n. d.
#19365	SAH	13	Positive	n. d.	n. d.
#17292	AF	17	Positive	n. d.	n. d.
#15040	AF	n. d.	n. d.	n. d.	n. d.
#17291	AF	15	Positive	8.9	Negative
#30/03	AF	4	Negative	1	Negative
#73/05	AF	17	Positive	13	Positive

#17293	AF	0	Negative	0	Negative
#14673	AF	15	Positive	10	Weakly positive
#13512	AF	18	Positive	12	Positive
#82/06	AF	16	Positive	3	Negative
#15159	AF	1	Negative	0	Negative
T#2	AF	22	Positive	n. d.	n. d.
#17065	SZ	1	Negative	0	Negative
#19100	SZ	n. d.	n. d.	12	Positive
#55/04	SZ	n. d.	n. d.	5	Negative
#16362	SZ	0	Negative	0	Negative
#13914	SZ	n. d.	n. d.	9.9	Weakly positive
#17266	SZ	13	Positive	10	Weakly positive
T#1	VZV meningitis	14	Positive	18	Positive
#14689	Normal pressure hydrocephalus	n. d.	n. d.	9	Weakly positive
#17442	Surgical hemorrhage, Glioblastoma °IV	n. d.	n. d.	n. d.	n. d.
T#3	Dissociative disorder	n. d.	n. d.	n. d.	n. d.
T#4	Multiple sclerosis	11	Positive	10	Weakly positive
T#5	Facial palsy, VZV	n. d.	n. d.	n. d.	n. d.
T#6	Facial palsy	14	Positive	10.9	Weakly positive
#17345	Hemophagocytic lymphohistiocytosis (HLH)	4	Negative	2	Negative

Table S4. HHV-6 serum serology. IgG and IgM positivity against HHV-6 is given for selected serum samples from *n* = 4 AF, *n* = 2 SZ, and *n* = 1 HLH patients. Serological testing was performed as described in Section 2; AF: Affective spectrum disorder; HHV-6: *human herpesvirus 6*; SZ: Schizophrenic spectrum disorder

Pat. ID.	Diagnosis	HHV-6 IgG plasma	HHV-6 IgM plasma
#17291	AF	Positive (1.33)	Negative (0.21)
#30/03	AF	Weakly positive (1.00)	Negative (0.28)
#17293	AF	Positive (1.10)	Negative (0.22)
#15159	AF	Positive (1.38)	Negative (0.32)
#17065	SZ	Positive (1.10)	Negative (0.28)
#16362	SZ	Negative (0.84)	Positive (6.75)
#17345	Hemophagocytic lymphohistiocytosis (HLH)	Negative (0.56)	Negative (0.10)

Table S5. Positively detected viral miRNA in each patients' groups. For HSV-1-derived miRNAs miR-H27, miR-H4-3p, miR-H3-3p, miR-H6-3p, miR-H2-3p, and miR-H4-5p, the numbers of positive samples (against all tested samples) are displayed for *n* = 5 controls (Ctrl), and patients with SAH (*n* = 5), AF (*n* = 11), SZ (*n* = 6), and various other neuroinflammatory diseases (*n* = 8). * Not all patients' samples not tested for every miRNA target (due to limited material). The cut-off for positive detection of viral miRNAs was set at a CT value of 40. An overview on all CT values is provided in Table S7.

**Group**	***hsv1*-miR-H2-3p**	***hsv1*-miR-H3-3p**	***hsv1*-miR-H4-3p**	***hsv1*-miR-H4-5p**	***hsv1*-miR-H6-3p**	***hsv1*-miR-H27**
Non-neuroinflammatory controls (Ctrl, *n* = 5)	0/5	3/3*	5/5	5/5	5/5	4/4*
Subarachnoid hemorrhage (SAH, *n* = 5)	4/5	5/5	4/5	4/5	5/5	5/5
Affective spectrum disorder (AF, *n* = 11)	7/11	11/11	6/11	8/11	10/10*	8/8*
Schizophrenic spectrum disorder (SZ, *n* = 6)	2/6	6/6	4/6	3/6	5/5*	6/6
Various other neuroinflammatory diseases (*n* = 8)	6/8	8/8	7/8	6/8	7/7*	7/7*

*Not all samples tested for selective *hsv1*-miRNA target (limited material)

Table S6. Candidate housekeeping gene comparisons. Relative miRNA stability was determined by the comparative ΔCT algorithm [38], as described in Section 2. The algorithm is based on the pair-wise comparison of candidate genes for all investigated samples, and derives the stability from the standard deviation of ΔCT values [38]. Mean ΔCT values are given as the mean difference between the CT values of the two respective miRNAs over all investigated samples. Standard deviation (StdDev) is given for the variation in CT values between the two respective miRNAs over all investigated samples. Mean StdDev values indicate the relative stability for ranking every miRNA as a potential housekeeping gene (see Figure S3). Calculation included a total of n = 33 samples for *hsv1*-miR-H3-3p, n = 32 samples for *hsv1*-miR-H6-3p, and n = 30 samples for *hsv1*-miR-H27. Viral miR-H2-3p, miR-H4-3p, and miR-H4-5p were excluded, since these miRNAs were frequently negative throughout all patient groups (see Table S5). An overview on all CT values is provided in Table S7.

**Target comparison**	**Mean ΔC$_T$**	**StdDev**	**Mean StdDev**
hsv1-miR-H3-3p vs. *hsv1*-miR-H6-3p	3.15	2.08	2.27
hsv1-miR-H3-3p vs. *hsv1*-miR-H27	3.29	2.45	
hsv1-miR-H6-3p vs. *hsv1*-miR-H3-3p	3.15	2.08	1.61
hsv1-miR-H6-3p vs. *hsv1*-miR-H27	1.79	1.13	
hsv1-miR-H27 vs. *hsv1*-miR-H3-3p	3.29	2.45	1.79
hsv1-miR-H27 vs. *hsv1*-miR-H6-3p	1.79	1.13	

Table S7: CT values for viral miRNAs in CSF-derived exosomes. CT values for HSV-1-derived miR-H2-3p (total *n* = 35 samples), miR-H3-3p (total *n* = 33 samples), miR-H4-3p (total *n* = 35 samples), miR-H4-5p (total *n* = 35 samples), miR-H6-3p, (total *n* = 32 samples) and miR-H27 (total *n* = 30 samples) are displayed for each sample of each patient cohort. Values are presented as mean values over three technical replicates. Due to limited material, some targets could not be determined for individual samples (n. d. = not determined); AF: Affective spectrum disorder; Ctrl: non-neuroinflammatory controls; HLH: hemophagocytic lymphohistiocytosis; NPH: Normal pressure hydrocephalus; SAH: Subarachnoid hemorrhage; SZ: Schizophrenic spectrum disorder; VZV: varicella zoster virus.

Pat. ID.	Diagnosis	CT *hsv1*-miR-H2-3p	CT *hsv1*-miR-H3-3p	CT *hsv1*-miR-H4-3p	CT *hsv1*-miR-H4-5p	CT *hsv1*-miR-H6-3p	CT *hsv1*-miR-H27
#17065	SZ	40,00	28,11	38,22	36,13	31,88	32,74
#19100	SZ	36,79	27,65	40,00	40,00	35,39	33,75
#55/04	SZ	35,08	29,42	40,00	33,60	n. d.*	30,79
#16362	SZ	40,00	31,62	37,39	40,00	34,66	32,89
#13914	SZ	40,00	30,23	37,00	40,00	33,65	31,95
#17266	SZ	40,00	31,01	37,13	36,95	35,88	35,25
#17292	AF	40,00	27,46	39,91	36,00	35,53	36,50
#15040	AF	40,00	27,26	39,57	37,24	33,60	35,88
#17291	AF	40,00	29,85	40,00	36,05	n. d.*	36,44
#30/03	AF	36,22	29,72	40,00	40,00	31,93	35,22
#73/05	AF	37,02	29,06	40,00	36,34	32,13	n. d.*
#17293	AF	38,91	31,41	37,51	36,59	35,64	34,78
#14673	AF	38,52	31,21	40,00	35,84	31,56	32,84
#13512	AF	40,00	32,77	40,00	36,33	29,48	n. d.*
#82/06	AF	32,14	31,21	32,32	31,45	27,63	n. d.*
T#2	AF	37,58	30,21	36,97	40,00	34,59	32,14
#15159	AF	36,97	31,21	35,52	40,00	33,47	31,94
T#1	VZV meningitis	36,33	30,59	35,26	40,00	32,59	31,42
T#3	Dissociative disorder	40,00	30,89	36,42	40,00	35,27	33,94
T#4	MS	40,00	30,34	35,04	37,02	34,11	31,03
T#5	VZV-associated facial palsy	38,04	30,96	38,46	35,74	33,39	33,45
#14689	NPH	36,51	27,48	37,02	36,39	34,51	33,47
#17442	Surgical hemorrhage, Glioblastoma °IV	37,42	27,48	36,62	37,02	29,95	31,46
#17345	HLH	37,64	31,69	40,00	35,95	n. d.*	n. d.*

T#6	Facial palsy	35,13	30,99	36,43	37,68	32,84	31,13
#19442	SAH	35,78	30,61	35,27	35,13	31,24	28,27
#19384	SAH	33,11	30,32	33,43	32,33	28,94	26,11
#18858	SAH	36,09	30,76	36,75	33,72	29,48	28,95
#14690	SAH	35,66	30,33	35,02	36,17	31,13	29,63
#19365	SAH	40,00	34,06	40,00	40,00	35,01	33,75
TK1	Ctrl	40,00	31,44	36,91	37,06	28,62	n. d.*
TK2	Ctrl	40,00	31,07	36,45	36,36	31,17	35,59
TK3	Ctrl	40,00	n. d.*	35,57	36,33	35,80	36,37
TK4	Ctrl	40,00	n. d.*	37,81	34,63	30,61	35,34
TK5	Ctrl	40,00	30,41	37,51	35,80	32,51	34,57

* Due to limited material, sample could not be tested

Table S8: Correlation of host- and HSV-1-derived miRNAs with NfL and IL-8. Fold changes of viral miR-H27, miR-H3-3p and host-derived miR-155-5p, miR-138-5p, miR-21-5p, and miR-146a-5p (see Figure 4) are correlated with CSF concentrations of NfL and IL-8 (see Figure 5). r: Spearman correlation coefficient, *p*: significance, Significant differences are marked by * $p \leq 0.05$, ** $p \leq 0.01$, and *** $p \leq 0.001$.

Correlation	Spearman r	Number of samples	*p* value
miR-H27 vs. NfL	0,43	26	0,03*
miR-155-5p vs. NfL	0,22	32	0,22
miR-H3-3p vs. NfL	0,01	28	0,98
miR-138-5p vs. NfL	0,37	32	0,04*
miR-21-5p vs. NfL	0,18	32	0,32
miR-146a-5p vs. NfL	-0,001	32	0,98
miR-H27 vs. IL-8	0,31	26	0,12
miR-155-5p vs. IL-8	0,30	32	0,09
miR-H3-3p vs. IL-8	-0,16	28	0,41
miR-138-5p vs. IL-8	-0,14	32	0,44
miR-21-5p vs. IL-8	0,48	32	0,01**
miR-146a-5p vs. IL-8	0,32	32	0,08

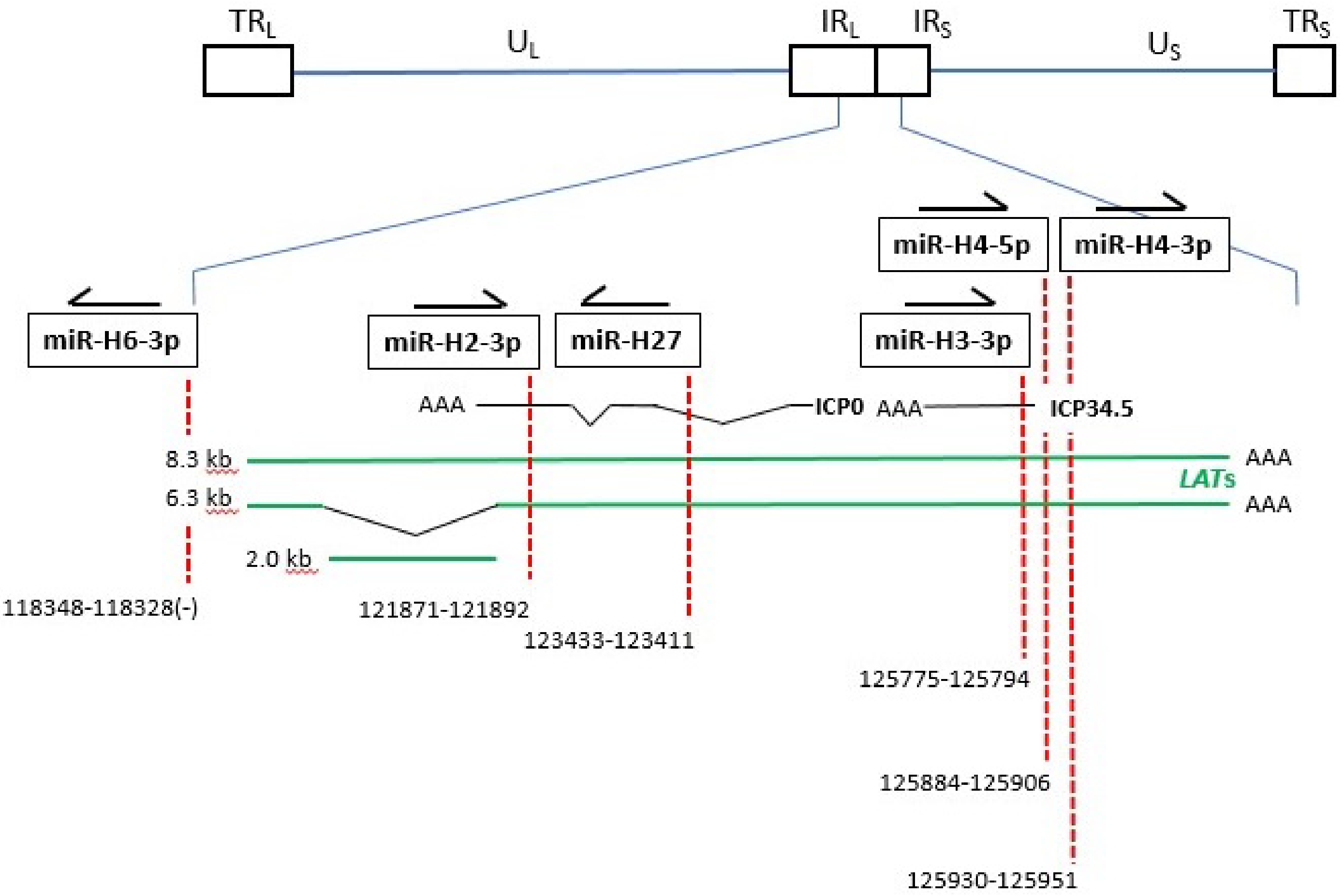

Figure S1. Selected HSV-1-derived miRNAs and their location within the HSV-1 genome. MiRNAs miR-H2-3p, miR-H3-3p, miR-H4-3p, miR-H4-5p, miR-H6-3p, and miR-H27 are shown by their location inside or outside of the HSV-1 genome-derived latency-associated transcripts (LATs; green lines, splice regions indicated by kinks). Dotted red lines indicate overlaps with coding regions of infected cell proteins (ICPs) ICP0 and ICP34.5, respectively (black lines, splice regions are indicated by kinks). Arrows on boxes indicate the coding region of the respective miRNA to be sense (right direction) or antisense (left direction) to the LAT. TRL: long terminal repeat; UL: long unique region; IRL: long internal repeat; IRS: short internal repeat; US: short unique region; TRS: short terminal repeat.

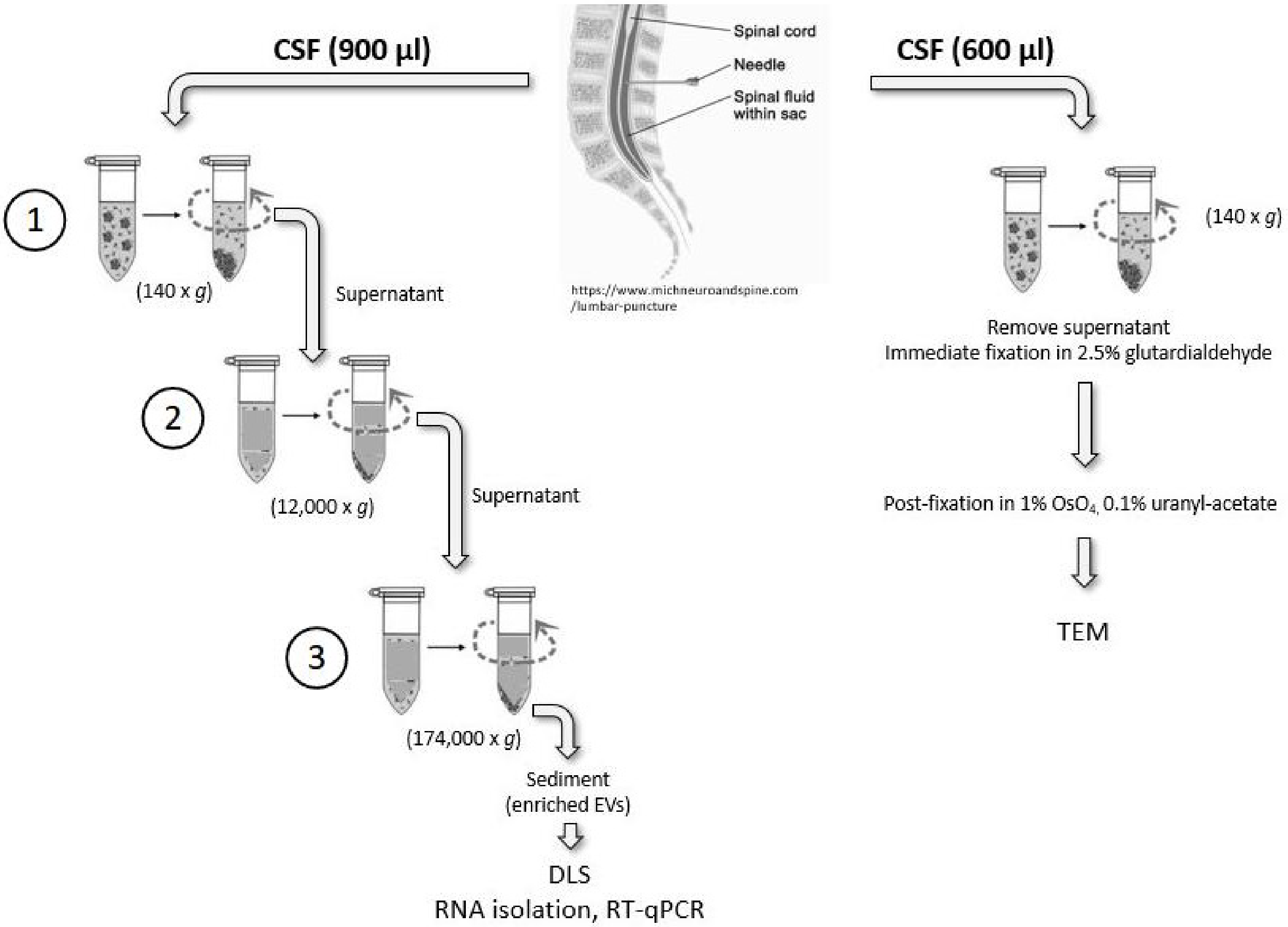

Figure S2. Overview on experimental procedure as described in Section 2. The left panel shows the centrifugation steps used for the enrichment of exosomes. Step 1 is a low speed centrifugation by a swing-out rotor to remove leukocytes. Step 2 is a higher centrifugation step (12,000 x *g*) performed in a fixed-angle rotor of an Eppendorf centrifuge to remove larger EVs derived from cell fragments, EVs from the plasma membrane, and apoptotic bodies. Step 3 is performed by ultracentrifugation using a Beckman Optima™ MAX-E (TLA 55 fixed-angle rotor) device. The sediment of Step 3 is subjected to DLS, RNA isolation, and qPCR. The right panel explains how leukocytes from CSF are enriched by a centrifugation, identical to step-1 centrifugation. After careful removal of the supernatant, the sedimented cells are fixed in 2.5% glutardialdehyde in PBS and further processed by postfixation with osmium teroxide (OsO4) and uranylaetate (UA) for ultrastructural analysis.

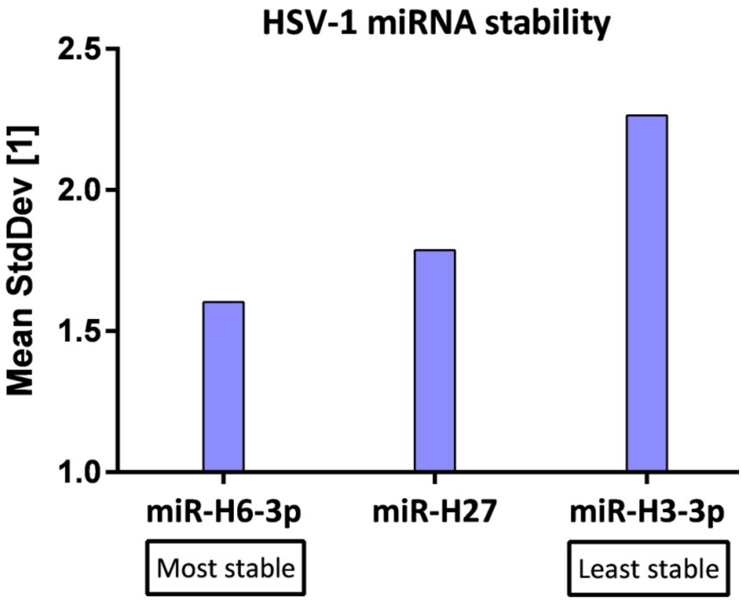

Figure S3. Viral miRNA stability. Stability of viral miRNAs was determined according to the comparative ΔCT algorithm [38], as described in Section 2. After pair-wise comparison of all viral miRNAs, the mean standard deviation (Mean StdDev) of ΔCT values (see Table S6) was plotted to determine the relative stability of the respective miRNA. Calculation included a total of n = 33 samples for *hsv1*-miR-H3-3p, n = 32 samples for *hsv1*-miR-H6-3p, and n = 30 samples for *hsv1*-miR-H27. Viral miR-H2-3p, miR-H4-3p, and miR-H4-5p were excluded, since these miRNAs were frequently negative throughout all patient groups (see Table S5). An overview on all CT values is provided in Table S7.

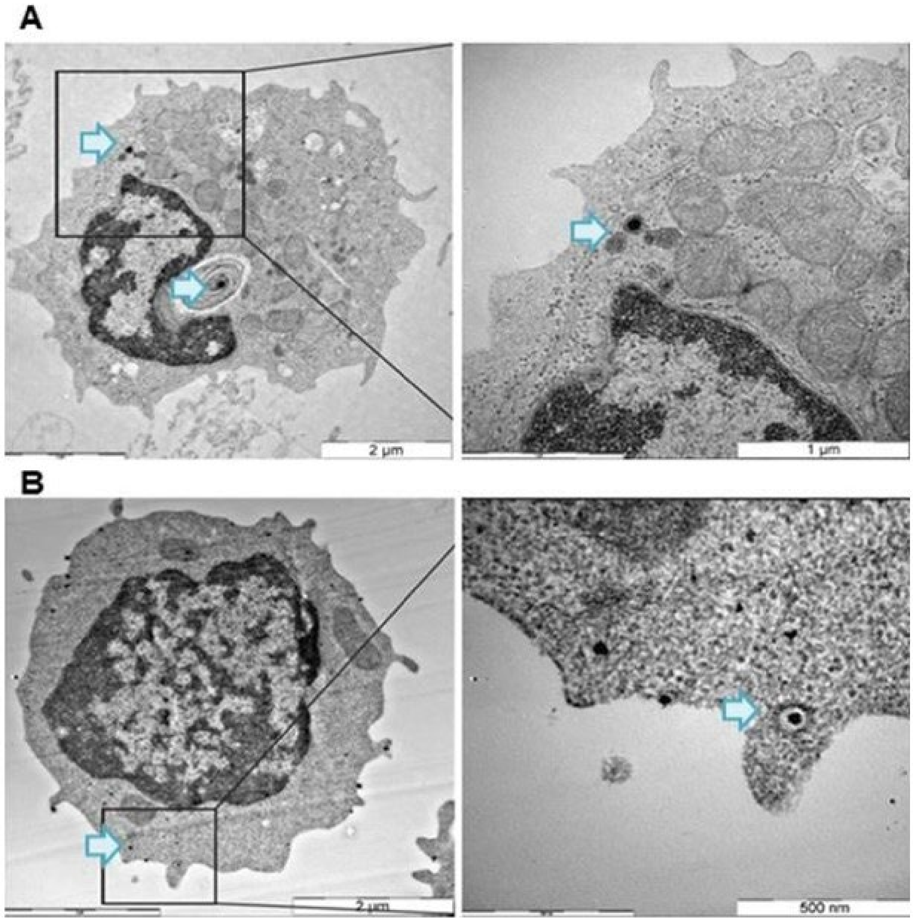

Figure S4. TEM images from CSF cell preparations of two AF patients. Upper row (A) shows a mitochondria-rich monocyte with a prominent multilamellar body and a central electron-dense particle and another electron-dense particle close to the plasma membrane (blue arrows) (A). Lower row (B) shows a lymphocyte with a likely virus particle in the cytoplasm with a highly electron-dense nucleocapsid surrounded by a light ring-shaped area and a more dense almost mature envelope. The characteristic asymmetry of the nucleocapsid is likely a herpesvirus.

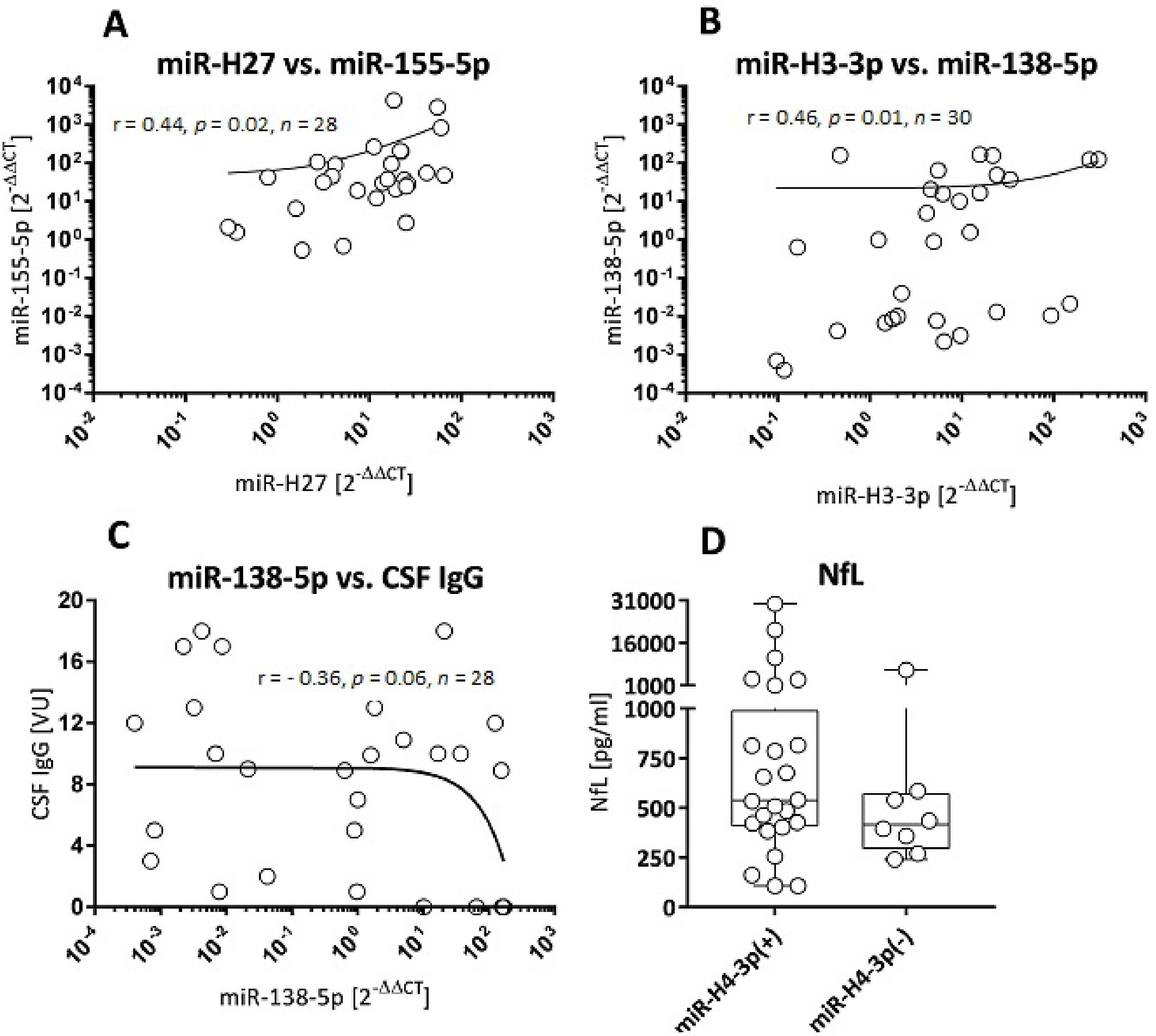

Figure S5. HSV-1- and host-derived miRNA expression. (A) shows the correlation between *n* = 28 *hsv1*-miR-H27 and *hsa*-miR-155-5p fold changes for all patients (see Figure 4). (B) shows the correlation between *n* = 30 *hsv1*-miR-H3-3p and *hsa*-miR-138-5p fold changes for all patients (see Figure 4). (C) shows the correlation between *n* = 28 *hsa*-miR-138-5p fold changes (see Figure 4) and HSV-1-directed CSF IgG titers (VU) for all patients. (D) presents CSF NfL levels for all patients, grouped into miR-H4-3p-positive (+) (*n* = 24) and -negative (-) (*n* = 8) specimen. Comparative measurements of NfL are presented as scatter plots; r: Spearman correlation coefficient, *p*: significance, *n*: number of specimen.

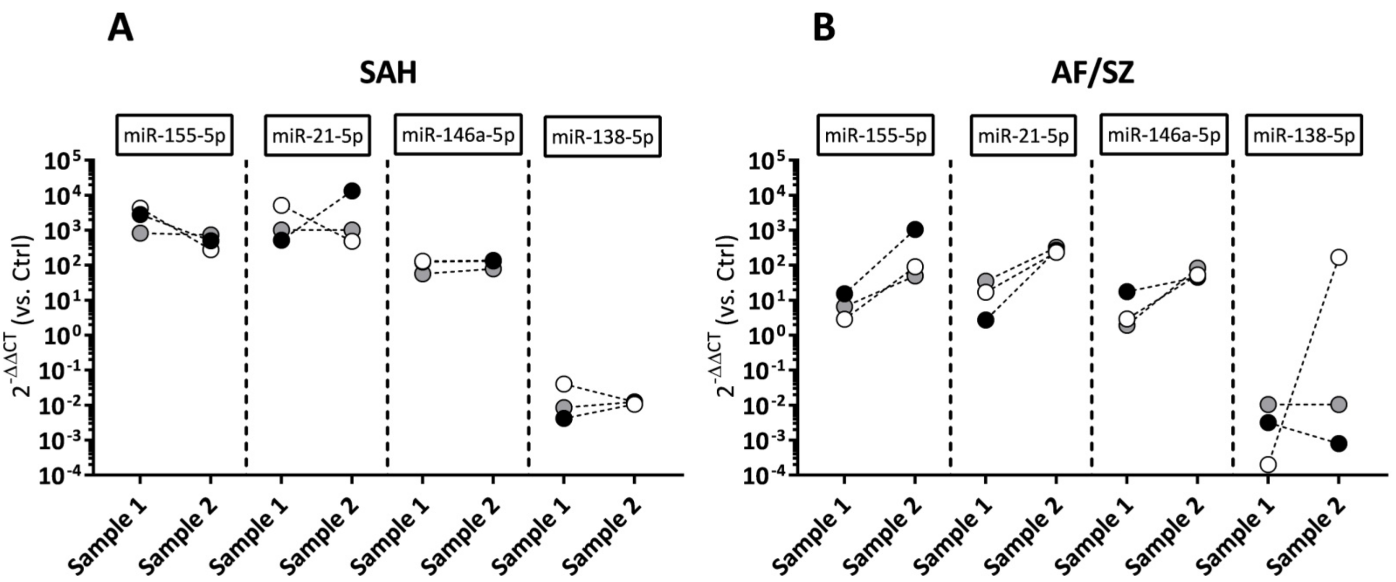

Figure S6. MiRNA profiles from CSF-derived exosomes for follow-up samples. Fold changes of miR-155-5p, miR-21-5p, miR-146a-5p, and miR-138-5p are presented for $n = 3$ selected SAH (A), and $n = 3$ selected psychiatric patients (2x AF, 1x SZ, B) in two follow-up CSF samples per patient. Fold changes were calculated using the $2^{-\Delta\Delta CT}$ method as described in *Materials and Methods*. Non-neuroinflammatory controls (Ctrl) served as the reference group, and spiked-in *cel*-miR-39-3p served as exogenous normalization control. Individual patients were color-labelled for SAH (black circle: patient #19384, open circle: patient #19365, grey circle: patient #19442; see detailed clinical data in Table S2) and psychiatric patients (black circle: AF patient #73/05, grey circle: AF patient #15040, open circle: SZ patient #17065). Time between CSF sampling was as follows, for SAH (patient #19384: 7 days, patient #19365: 4 days, patient #19442: 6 days) and psychiatric patients (AF patient #73/05: 6 months, AF patient #15040: 3 months, SZ patient #17065: 6.5 months).
